# Rapid AST in practice – a workflow analysis of the QuickMIC^®^ rapid AST system at multiple clinical laboratories in Europe

**DOI:** 10.3389/fcimb.2026.1823965

**Published:** 2026-05-05

**Authors:** Amanda Kindvall Åhman, Victor Englöf, Kajsa Knagge, Lucas Reibenspies, Maria Avolio, Patricia Cabral da Silva, Patrizia Cambieri, Cristina Costa, Adriana Coutinho, Monica Dotta, Pier Andrea Dusi, Danielle Fenwick, Giorgia Gregori, Sandra Grunewald, Kerry Laws, Giuliana Lo Cascio, Paulo Lopes, Emma Marrs, Luis Morais, Joni Mota, Loredana Pangaro, Micaela Pelagi, Sara Petersson, Damiano Picicco, Tamara Ruegamer, Sarah Shodunke, Annika Wistedt, Hanna Woksepp, Andrea Zappavigna, Giuliana Germinario, Alessia Cantiani, Simone Ambretti, Cecilia Johansson, Anna Olsson, Christer Malmberg

**Affiliations:** 1 Gradientech AB, Uppsala, Sweden; 2 Microbiology and Virology Unit, University Hospital Città della Salute e della Scienza di Torino, Turin, Italy; 3 Department of Clinical Microbiology, Kalmar County Hospital, Kalmar, Sweden; 4 Struttura Complessa (SC) Microbiologia e Virologia, Fondazione IRCCS Policlinico San Matteo, Pavia, Italy; 5 Laboratório de Patologia Clínica, Hospital do Espírito Santo de Évora, Évora, Portugal; 6 Struttura Semplice Dipartimentale (SSD) Microbiologia, Ospedale di Sanremo, Sanremo, Italy; 7 Microbiology Research, Freeman Hospital, Newcastle upon Tyne, United Kingdom; 8 Dipartimento Medicina di Laboratorio, Azienda Unità Sanitaria Locale (AUSL) di Piacenza, Ospedale Guglielmo da Saliceto, Piacenza, Italy; 9 Institute for Medical Microbiology, Immunology and Hygiene, University Hospital Cologne, Cologne, Germany; 10 Faculty of Medicine, University of Cologne, Cologne, Germany; 11 Department of Microbiology of Clinical Pathology, Unidade Local de Saúde Gaia Espinho, Vila Nova de Gaia, Portugal; 12 Department of Microbiology of Clinical Pathology, Unidade Local de Saúde do Médio (ULSM) Tejo, Tomar, Portugal; 13 Department of Clinical Microbiology, Vercelli Hospital, Vercelli, Italy; 14 Department of Biomedical and Clinical Sciences, Linköping University, Linköping, Sweden; 15 Department of Medical and Surgical Sciences, Alma Mater Studiorum - University of Bologna, Bologna, Italy; 16 Microbiology Unit, IRCCS Azienda Ospedaliero Universitaria di Bologna, Bologna, Italy; 17 Department of Medical Sciences, Uppsala University, Uppsala, Sweden

**Keywords:** antimicrobial resistance, diagnostics, microfluidic, rapid AST, sepsis

## Abstract

**Objectives:**

Timely administration of appropriate antimicrobial therapy is critical for sepsis management, yet conventional antimicrobial susceptibility testing (AST) methods typically provide results within 24–48 hours after positivity. We evaluated the performance and workflow impact of a novel rapid AST system that delivers actionable results within 2–4 hours, compared to standard in-house laboratory methods used across multiple European centers.

**Methods:**

A prospective, multicenter study was conducted at 12 hospitals in the United Kingdom, Germany, Sweden, Italy, and Portugal. Positive blood culture samples were analysed in parallel using the QuickMIC rapid AST system and the local reference AST method (broth microdilution, automated systems, rapid AST, or disk diffusion, depending on site). Essential agreement, categorical agreement, and error rates were calculated as compared to the local method. In addition, workflow analysis quantified the time saved in delivering actionable AST results, defined as the availability of clinically interpretable susceptibility categories.

**Results:**

For a total of 306 evaluable isolates (from 309 collected) across all hospitals, QuickMIC demonstrated high concordance with reference methods, with categorical agreement exceeding 90% for the majority of tested antibiotic–pathogen combinations. Workflow analysis revealed a potential reduction in turnaround time of 17–45 hours compared with standard methods. For 88% of all tests, this allowed actionable AST results to be available within the same clinical shift rather than on subsequent days. This acceleration was consistent across both northern and southern European sites despite heterogeneity in reference methods and local laboratory workflows.

**Conclusions:**

The novel rapid AST system reliably provides susceptibility results in 2–4 hours directly from positive blood cultures, with performance comparable to established in-house methods. Implementation of this approach can substantially shorten the time to actionable results, offering clinicians the ability to optimize antimicrobial therapy significantly earlier during sepsis management. These findings support the adoption of rapid AST as a valuable tool to improve patient care and stewardship outcomes across diverse European hospital settings.

## 1 Introduction

Effective antimicrobial therapy is especially critical for managing patients with bacteremia, sepsis or septic shock (Bauer et al., 2020; Okeke et al., 2024). Since the global incidence of sepsis in 2017 was estimated to approximately 48.9 million cases, with a mortality rate of 32.5% (Bauer et al., 2020; Ikuta et al., 2022), increasing AMR could lead to severe consequences in this patient population. Cases of AMR-related sepsis have been steadily increasing over the past several decades, with AMR contributing to around 35% of sepsis deaths in 2019 (Naghavi et al., 2024). As AMR continues to increase, current empirical treatments will become increasingly ineffective. As a result, available effective antimicrobial treatments will be limited, affecting patients in need for prophylactic and/or therapeutic antimicrobial interventions, resulting in heightened mortality and morbidity of critical disease such as bloodstream infections (Ibrahim et al., 2000; Kollef, 2000; Marston et al., 2016).

To mitigate the growing threat of AMR, new strategies leading to a decrease in empirical, broad-spectrum antibiotic usage is crucial (Marston et al., 2016). Since the introduction of new antimicrobials is not as fast as the occurrence of new resistant pathogens, implementation of control measures, policies, and targeted antibiotic treatment based on susceptibility testing is necessary (Laxminarayan et al., 2024). Rapid antibiotic susceptibility testing (rapid AST) is a promising strategy to quickly define the susceptibility profile for the bacterial agent (van Belkum and Dunne, 2013; Vasala et al., 2020). This allows administration of a more personalized and targeted antibiotic treatment, while reducing the unnecessary use of broad-spectrum antibiotics. Importantly, rapid AST facilitates faster escalation of therapy when inappropriate empirical treatments are used, which is closely linked to treatment outcome by multiple studies showing that every hourly delay of appropriate antimicrobial therapy after arrival to the hospital increases mortality (Leibovici et al., 1998; Liu et al., 2017; Hogan et al., 2020).

**Figure 1:**
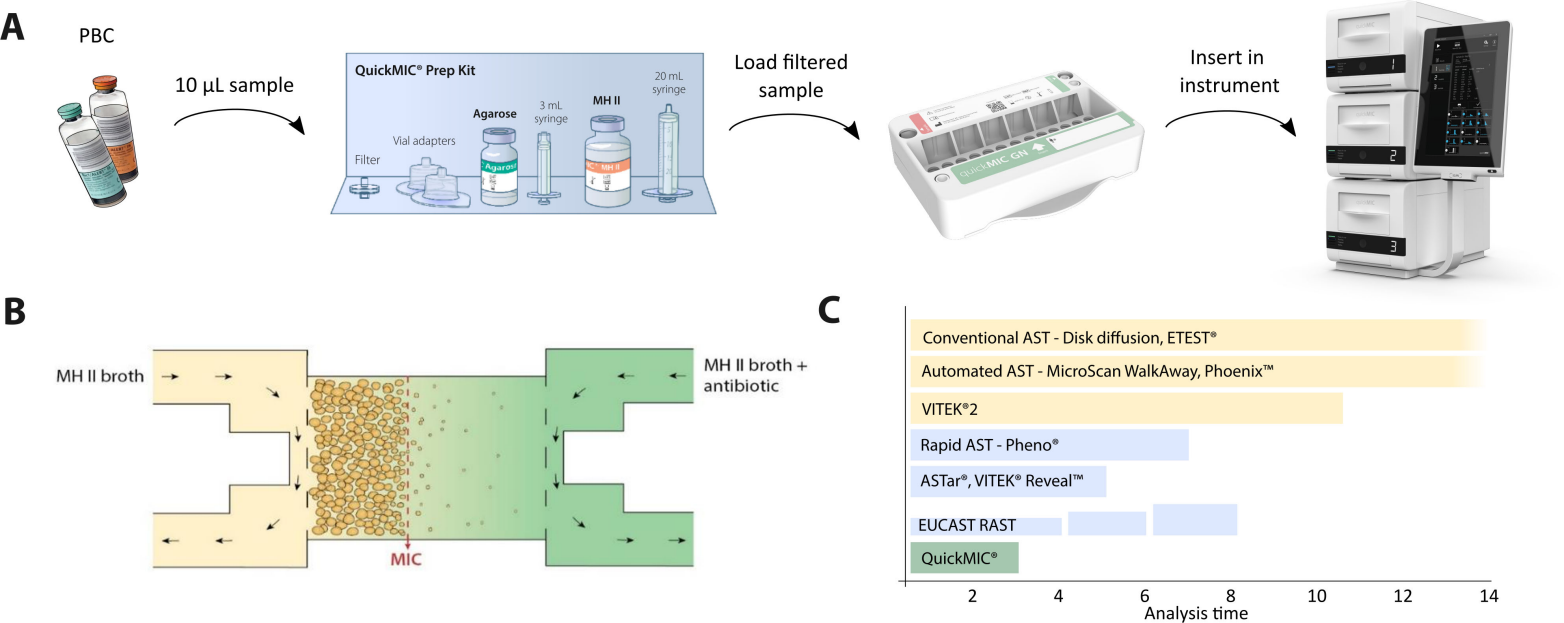
Overview of the QuickMIC rapid AST system. **(A)** Schematic workflow of rapid AST directly from positive blood-culture (PBC), where 10 µL of PBC is diluted in an agarose vial and filtered before injection in the GN cassette and loaded into the QuickMIC instrument for analysis. **(B)** Detail of a test chamber on the QuickMIC GN cassette, where a linear antibiotic gradient is formed over the test chamber containing growing bacteria. **(C)** Comparison of test analysis times between QuickMIC ultra-rapid AST and standard test methods as well as rapid AST systems and the manual EUCAST RAST method. The EUCAST RAST method is read at 4, 6 and 8 hours, with an increasing number of readable zones by each timepoint.

Several rapid AST systems have been developed in the last decade, and a few have already been integrated into the routine clinical workflow such as EUCAST rapid disc diffusion (Jonasson et al., 2020). Multiple established automated AST systems are also in use, e.g. VITEK^®^ 2 and BD Phoenix™ Automated Microbiology system (Belkum et al., 2020). These can provide a faster susceptibility profile (<8 h-16 h) than the legacy routine methods (24–48 h). However, receiving the results within one work shift is desirable for enabling actionable measures in a significantly shorter time, which might indicate a need for even faster solutions (Idelevich and Becker, 2019). QuickMIC (Gradientech AB, Uppsala, Sweden) is a new ultra-rapid (<4 h) AST system that uses microfluidics to create a linear gradient of antibiotics (**Figure 1**). The QuickMIC GN cassette provides growth-based MIC-values for 12 antibiotics for Gram-negative bacteria within 2–4 hours directly from monomicrobial positive blood cultures. The performance of QuickMIC, e.g. reproducibility, trueness, precision and accuracy in comparison to reference broth microdilution (BMD) as well as other methods has previously been studied (Malmberg et al., 2022; Berinson et al., 2024; García-Rivera et al., 2024), but little information is available demonstrating the use of QuickMIC or other rapid AST systems in real-world situations. The aim for this study was to compare the QuickMIC ultra-rapid AST workflow to those of existing legacy AST methods, with emphasis on analysing how the earlier rapid AST result may affect the potential to earlier guided therapy in a wide variety of different laboratories and settings.

## 2 Material and methods

### 2.1 Study design

A method comparison (inter-method agreement) study was conducted at twelve European clinical microbiology laboratories between September 2023 to May 2025 to compare the QuickMIC ultra-rapid AST system to commonly used AST methods. In parallel, the value of an ultra-rapid AST system in the clinical diagnostic workflow of each laboratory was assessed by structured workflow analysis, where the participating laboratories answered a set of questions relating to local workflows. Monomicrobial Gram-negative bacteria from leftover positive blood-cultures (PBC) at each laboratory were analysed using the QuickMIC system as well as with the standard AST method used at each site, and the results were compared with respect to essential and categorical agreement (EA, CA) as well as bias. The study design was not a clinical study, no patients were enrolled, and all tested bacterial samples were fully anonymized and not traceable to any individual.

### 2.2 Structured workflow analysis

Interviews were performed with representatives from each laboratory, to collect data on the routine workflow for patients with confirmed bacteraemia or positive bacterial sepsis and confirmed Gram-negative bacteria. The workflow analysis included aspects such as opening hours and individual workflow steps, as well as typical timings based on expert estimate or retrospective analysis of the standard daily inflow of positive blood cultures. The full interview questionnaire can be seen in **Supplementary Table 1**. An overview of methods and protocols used at each site is found in **Table 1**. In general, after blood culture positivity, Gram-staining was performed, and blood cultures of Gram-negative origin were further subjected to species identification and AST. Commonly used AST methods were BD Phoenix™ (BD Biosciences), RAST/Disc diffusion (EUCAST), MicroScan WalkAway 96 plus (Beckman Coulter) or VITEK^®^ 2 (bioMérieux). All sites relied on MALDI-TOF for species identification, either MALDI Biotyper^®^ (Bruker) or Vitek-MS (bioMérieux). For QuickMIC testing, the manufacturers protocol was followed and all sites used QuickMIC^®^ GN (art. number: 43-001) for analysis. For the comparator methods, the methods were performed as per local protocol, which could be either according to the manufacturers instruction or a lab-developed protocol (direct from blood or rapid subculturing). Furthermore, the time-to-result (TTR) from analysis start of QuickMIC to the actionable result was compared to the turnaround-time (PBC processing to actionable result, TAT) times reported by each laboratory as well as to previously published TAT data for the analysis times of the methods in use at the laboratory.

**Table 1:** Details of the study locations and operating conditions. The partaking laboratories are anonymized with a study site number.

Study site	1	2	3	4	5	6	7	8	9	10	11	12
Location in Europe	South	South	South	South	South	North	South	South	South	North	North	South
Size (beds)	900	449	600	1 400	333	400	298	452	594	1511	1400	1374
Blood culture sets/y	20 000	10 300	25 000	57 000	6000	33 000	15 043	8250	44 000	33 000	35 000	40 000
Intensive care units	2	2	1	5	1	2	1	1	2	9	5	5
Antibiotic Stewardship Team	Yes	No	Yes	Yes	Yes	Yes	Yes	No	Yes	Yes	Yes	Yes
Opening hours of the laboratory	24/7 Daily	08–15 Mon-Sat	07:30–20 Mon-Fri, 07:30-14:30 Sat-Sun	8–20 Mon-Sat	08–16 Mon-Fri, 8–12 Sat-Sun	08–17 Mon-Fri, 10-13:30 Sat-Sun	08–20 Mon-Fri, 08–15 Sat and Emergency Lab 24/7	24/7 Daily	08-17h Daily	07.30-20:00 h Mon-Fri, 07.30-14:00 Sat-Sun	08:30–20 Mon-Fri. 07:30-12:30 Sat-Sun Emergency lab: 24/7.	24/7 Daily
AST service	During opening hours	During opening hours, before noon	During opening hours	During opening hours	During opening hours	During opening hours	During opening hours Mon-Sat	During opening hours	During opening hours	During opening hours	During opening hours	During opening hours
Blood culture system	BACTEC^™^ (BD)	BACTEC^™^ (BD)	BACT/ALERT^®^ VIRTUO^®^ (bioMérieux)	BACT/ALERT^®^ VIRTUO^®^ (bioMérieux)	BACTEC^™^ (BD)	BACT/ALERT^®^ VIRTUO^®^ (bioMérieux)	BACT/ALERT^®^ VIRTUO^®^ (bioMérieux)	BACTEC^™^ (BD)	BACT/ALERT^®^ (bioMérieux)	BACTEC^™^ (BD)	BACT/ALERT^®^ VIRTUO^®^ (bioMérieux)	BACTEC^™^ (BD)
AST system	Sensititre™ (Thermo Fisher Scientific, USA) BD Phoenix™ (Becton, Dickinson and Company, USA)	BD Phoenix™ (Becton, Dickinson and Company, USA)	VITEK^®^ 2 (bioMérieux, France)	MicroScan WalkAway 96 Plus (Beckman Coulter, USA)	VITEK^®^ 2 (bioMérieux, France)	RAST, EUCAST	VITEK^®^ 2 (bioMérieux, France)	VITEK^®^ 2 (bioMérieux, France, Accelerate Pheno)	VITEK^®^ 2 (bioMérieux, France)	VITEK^®^ 2 (bioMérieux, France)	EUCAST Disc Diffusion	MicroScan WalkAway 96 Plus (Beckman Coulter, USA), Merlin Broth Microdilution
Bacterial ID method	MALDI Biotyper^®^ (Bruker)	MALDI Biotyper^®^ (Bruker)	VITEK^®^ MS (bioMérieux, France)	MALDI Biotyper^®^ (Bruker)	VITEK^®^ MS (bioMérieux, France)	MALDI Biotyper^®^ (Bruker)	VITEK^®^ MS (bioMérieux, France)	MALDI Biotyper^®^ (Bruker)	VITEK^®^ MS (bioMérieux, France)	MALDI Biotyper^®^ (Bruker)	MALDI Biotyper^®^ (Bruker)	MALDI Biotyper^®^ (Bruker)

### 2.3 Data analysis

The published and self-reported TAT of each AST system was used to calculate the potential reduction in TAT from ultra-rapid AST instead of the standard system. The AST results from the individual systems were furthermore used to calculate EA, CA, bias, and fraction of minor error, major error and very major error in comparison to QuickMIC. EA and bias were calculated according to performance parameters defined by ISO 20776-2:2021. Fraction of errors was calculated per the entire dataset. EUCAST clinical breakpoints v15 (available at www.eucast.org) were applied to determine the susceptibility categories. Multi-drug resistance (MDR) was defined as resistant against at least three antibiotic classes: aminoglycosides (amikacin, gentamicin, tobramycin), cephalosporins (cefepime, ceftazidime, cefotaxime), and carbapenems (meropenem). The overall QuickMIC performance in comparison to all included methods was also calculated and split per-antibiotic to investigate systematic trends in QuickMIC performance. For statistical comparisons, Student’s t-test was used to compare means, and the limit for significance was set to p <0.05.

## 3 Results

### 3.1 Sample characteristics

**Figure 2:**
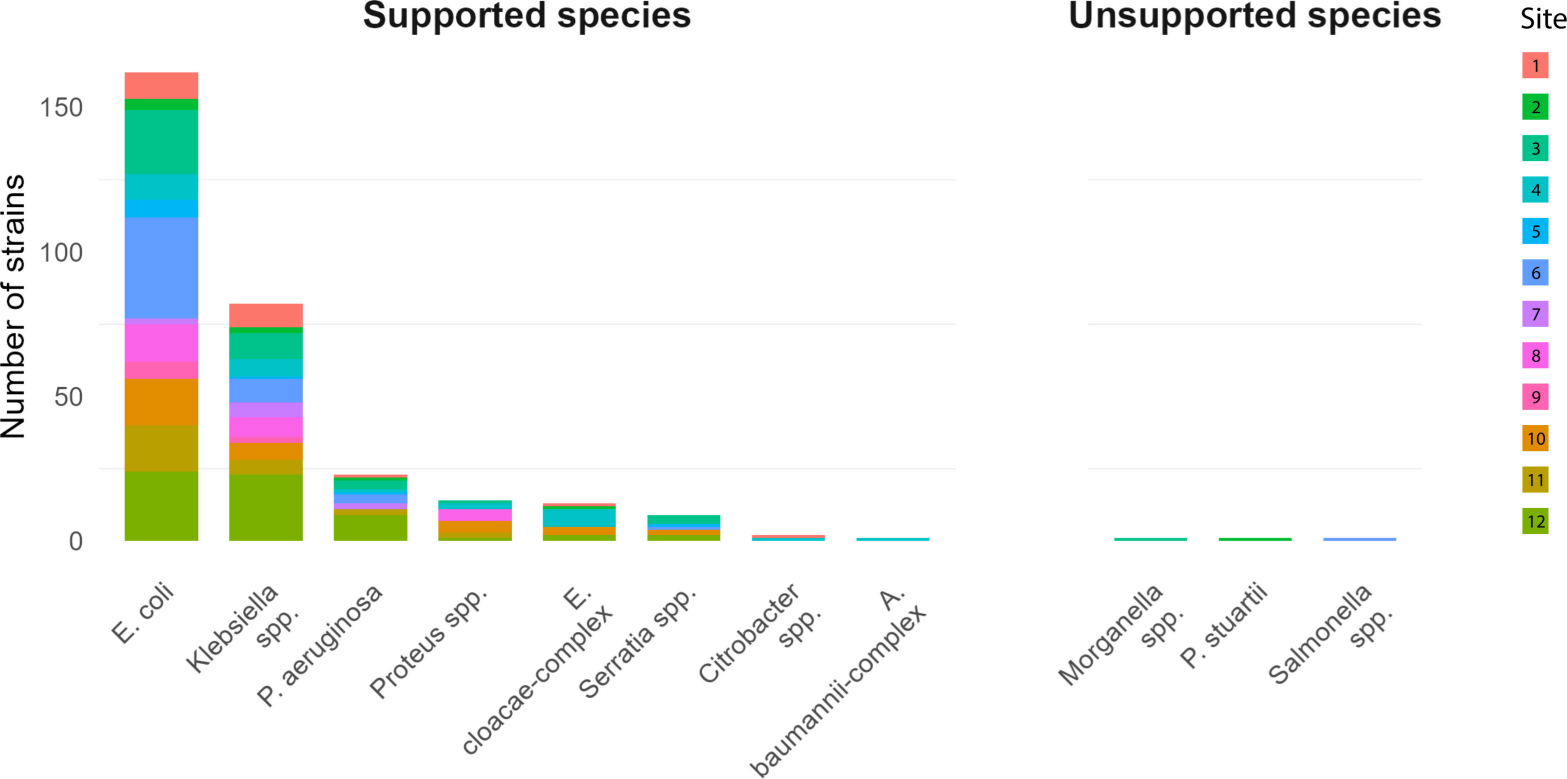
Distribution of species tested during the study period at each participating site. Sites are distinguished by color. In general, species distribution is similar between sites, and species incidence follows expected patterns for bloodstream infections with E. coli, Klebsiella spp. and P. aeruginosa being the 3 most common isolated species.

In total, 309 bacterial strains isolated from PBCs were analysed, with the number of bacterial isolates included from each site ranging between 8–61 per laboratory over the study duration (**Table 2**). Median study duration was 48 days (range 27–189). Active study periods ranged from 6 to 42 days, with activity defined as any day a QuickMIC run was performed. The difference in numbers of samples included per site reflect the differences in sample throughput per laboratory and number of days where the study was actively performed. As expected, *E. coli* was the most commonly detected bacterial species, followed by *K. pneumoniae-complex* and *P. aeruginosa* (**Figure 2**). The remaining species were either other Enterobacterales or *A. baumannii*, representing <15% of the total tested samples. Rates of MDR bacteria varied between the laboratories (0 – 55.6%, **Supplementary Table 2**), with a significantly higher incidence in laboratories in southern Europe (average 22.8% vs 5.8%, p <0.01). Overall, three (1.0%) samples yielded bacterial species not covered by the QuickMIC IFU (**Figure 2**).

**Table 2:** Study duration at each laboratory, and turnaround time (TAT) result statistics.

Site	First sample	Last sample	Total study duration (days)	Total samples (n)	Active days (n)	Within same shift results (n)	Within same shift results (%)
1	2024-03-28	2024-04-30	33	20	15	20	100.0
2	2024-05-20	2024-06-17	28	9	9	0	0.0
3	2023-09-27	2024-04-03	189	39	31	30	76.9
4	2024-07-04	2024-09-03	61	26	13	25	96.2
5	2024-02-29	2024-03-27	27	9	9	9	100.0
6	2024-03-07	2024-05-29	83	48	30	40	83.3
7	2024-01-10	2024-02-09	30	9	8	9	100.0
8	2024-04-11	2024-09-17	159	24	19	24	100.0
9	2024-06-19	2024-08-06	48	8	6	7	87.5
10	2024-07-15	2024-08-19	35	31	15	24	77.4
11	2025-03-03	2025-04-24	52	25	15	25	100.0
12	2024-11-08	2025-05-12	185	61	42	61	100.0
Overall	2023-09-27	2025-05-12	593	309	171	274	88.7

The number and percent of same shift results are calculated by the number of AST results which were ready for reporting within the AST service hours of the laboratory, compared to the total number of AST tests run.

### 3.2 Comparative performance

**Figure 3:**
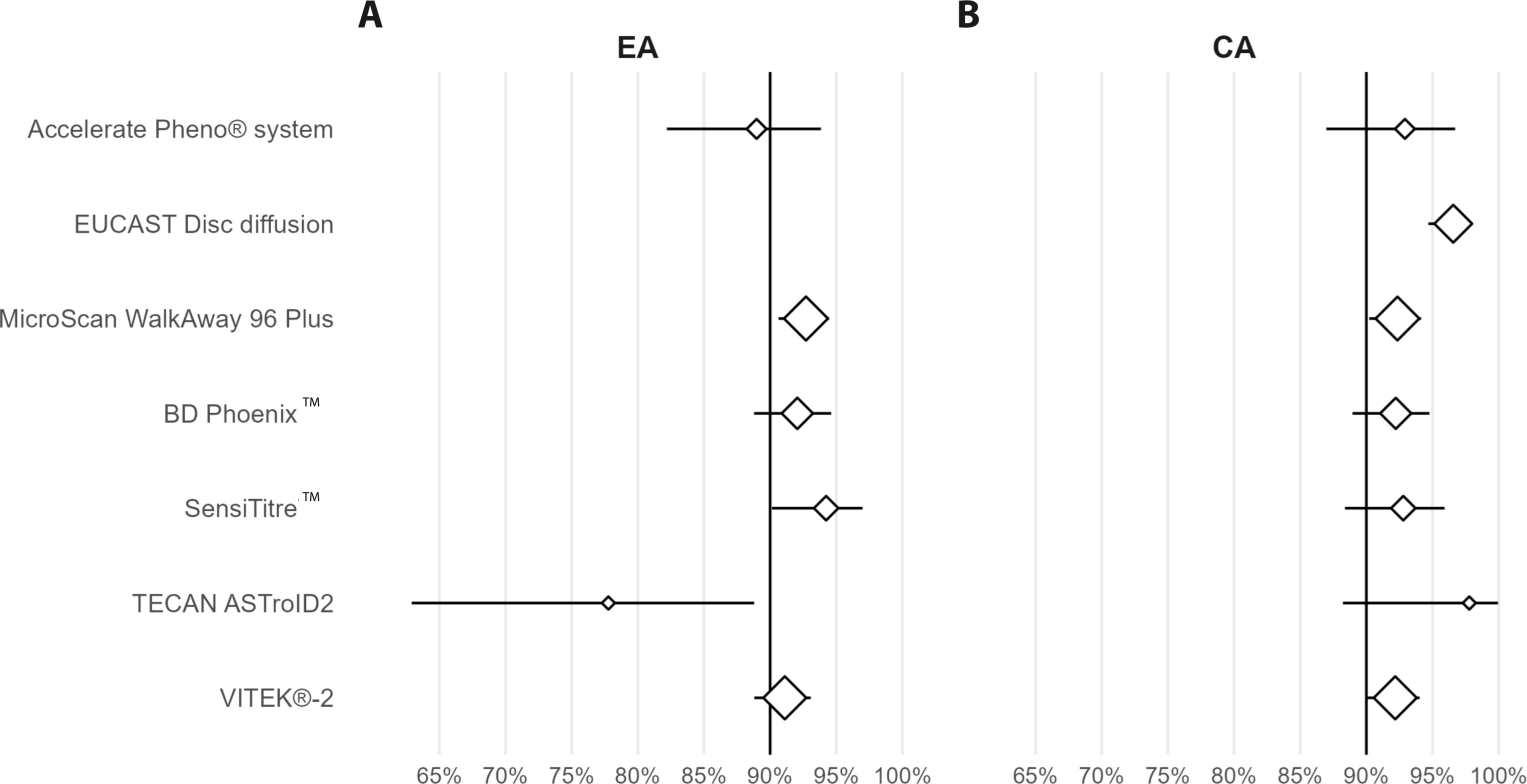
Forest plot showing the variation in A) EA and B) CA as compared to each routine method included in the study. The lines indicate 95% confidence intervals, and the points indicate the average agreement. The point size is proportional to the amount of datapoints for each method **(n)**. The 90% line is the accepted limit of agreement for EA and CA. In general, QuickMIC shows acceptable agreement as compared to the reference methods. Note that species distribution and sample size differ between the methods, and since no reference test was used the direction of discrepancies cannot be resolved.

The QuickMIC AST results were compared with each routinely used AST method, and in the case where several laboratories used the same method they were grouped. Breakdown of performance data per laboratory, species (supported and unsupported) and antibiotic tested can be found in **Supplementary Table 2**. Overall, QuickMIC agreement with traditional AST methods was good with an overall agreement of 93.3% and 91.7% for categorical and essential agreement, respectively (**Table 3**). While no single method had significantly poor comparative performance, the lowest EA was compared to the Accelerate Pheno rapid AST system and TECAN ASTroID (Merlin plates) automated AST platform (**Figure 3**). Even though it was not part of the study design, some laboratories investigated discrepancies using in-house broth microdilution. There was no systematic trend to be seen for which method best agreed with reference in this small and limited dataset (data not shown).

**Table 3:** Comparative performance of study site routine method versus QuickMIC with focus on agreements, errors and bias.

Method	No. of MIC	No. of categories	No. of strains	CA^1^	EA^1^	S	I	R	MiD^1^	MD^1^	VMD^1^	Bias	Time
Accelerate Pheno^®^ system	127	127	15	118 (92.9)	113 (89)	94	2	31	2 (1.6)	1 (0.8)	6 (4.7)	-12.00	03:08
EUCAST Disc diffusion	0	553	72	534 (96.6)	-	497	13	43	5 (0.9)	4 (0.7)	10 (1.8)	-	03:06
MicroScan WalkAway 96 Plus	768	758	79	700 (92.3)	712 (92.7)	578	20	160	22 (2.9)	11 (1.5)	25 (3.3)	-5.00	03:07
BD Phoenix™	365	360	45	332 (92.2)	336 (92.1)	229	16	115	12 (3.3)	3 (0.8)	13 (3.6)	-8.00	03:14
Sensititre™	208	208	30	193 (92.8)	196 (94.2)	167	8	33	5 (2.4)	1 (0.5)	9 (4.3)	-7.00	03:05
TECAN ASTroID^2^	45	45	8	44 (97.8)	35 (77.8)	17	27	1	1 (2.2)	0 (0)	0 (0)	19.00	02:47
VITEK^®^-2	731	729	119	672 (92.2)	666 (91.1)	568	33	128	27 (3.7)	12 (1.6)	18 (2.5)	-2.00	03:08
Overall	2,244	2,780	306	2593 (93.3)	2058 (91.7)	2,150	119	511	74 (2.7)	32 (1.2)	81 (2.9)	-4.37	03:07

^1^Values are n (%); ^2P^*P. aeruginosa* and *A. baumannii* only. No. of MIC: total number of MIC values generated. No. of categories: total number of interpreted susceptibility categories generated. No. of strain: total number of unique bacterial strains tested. CA: nr and percent test results within categorical agreement with reference result. EA: nr and percent test results within essential agreement with reference result. S, I, R: number of susceptible, increased exposure and resistant results by the reference method. MiD: minor error, MD: major error, VMD: very major error. Bias calculated as per ISO29776-2. Time: Time to result (TTR) for QuickMIC.

### 3.3 Time to results

**Figure 4:**
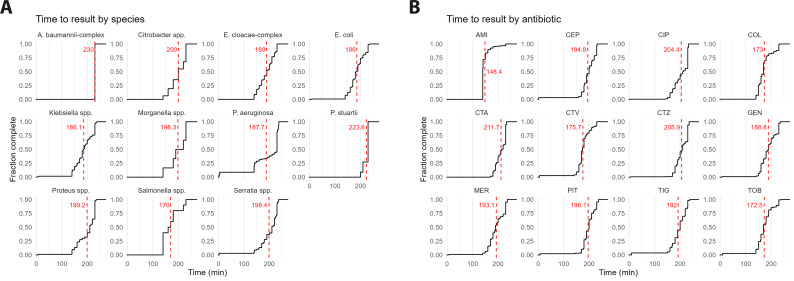
Cumulative time until stable MIC-value readout (time to result, TTR) for QuickMIC ultra-rapid AST for all species **(A)** and antibiotics **(B)** included. The average time to result is shown in red, with a dashed line and number of minutes. In general, there are no large differences between species and antibiotics, except for A. baumannii-complex with a significantly longer time to result.

QuickMIC consistently provided a MIC result at or below 4 h, with an average of 3 h and 7 min (range from 2 h 20 min to 4 h) over all study locations (**Figure 4**). Time to result (TTR) did not vary significantly among locations, however, the antibiotic and species tested were significant factors (p <0.001). For the included antibiotics, QuickMIC TTR varied per tested antibiotic on average from 2 h 31 min to 3 h 33 min (amikacin (AMI) and cefotaxime (CTA), respectively). Among the different species tested covered in the QuickMIC IFU, *E. coli* gave on average a result in 3 h 7 min, while *A. baumannii* was the slowest with an average of 3 h 22 min. When accounting for the AST service hours of each laboratory, QuickMIC provided a result in the same shift between 0 – 100% of all runs (**Table 2**; **Figure S5**), with an average of 88.7% of all runs providing a result on the same AST service hour shift.

### 3.4 Structured workflow analysis

**Figure 5:**
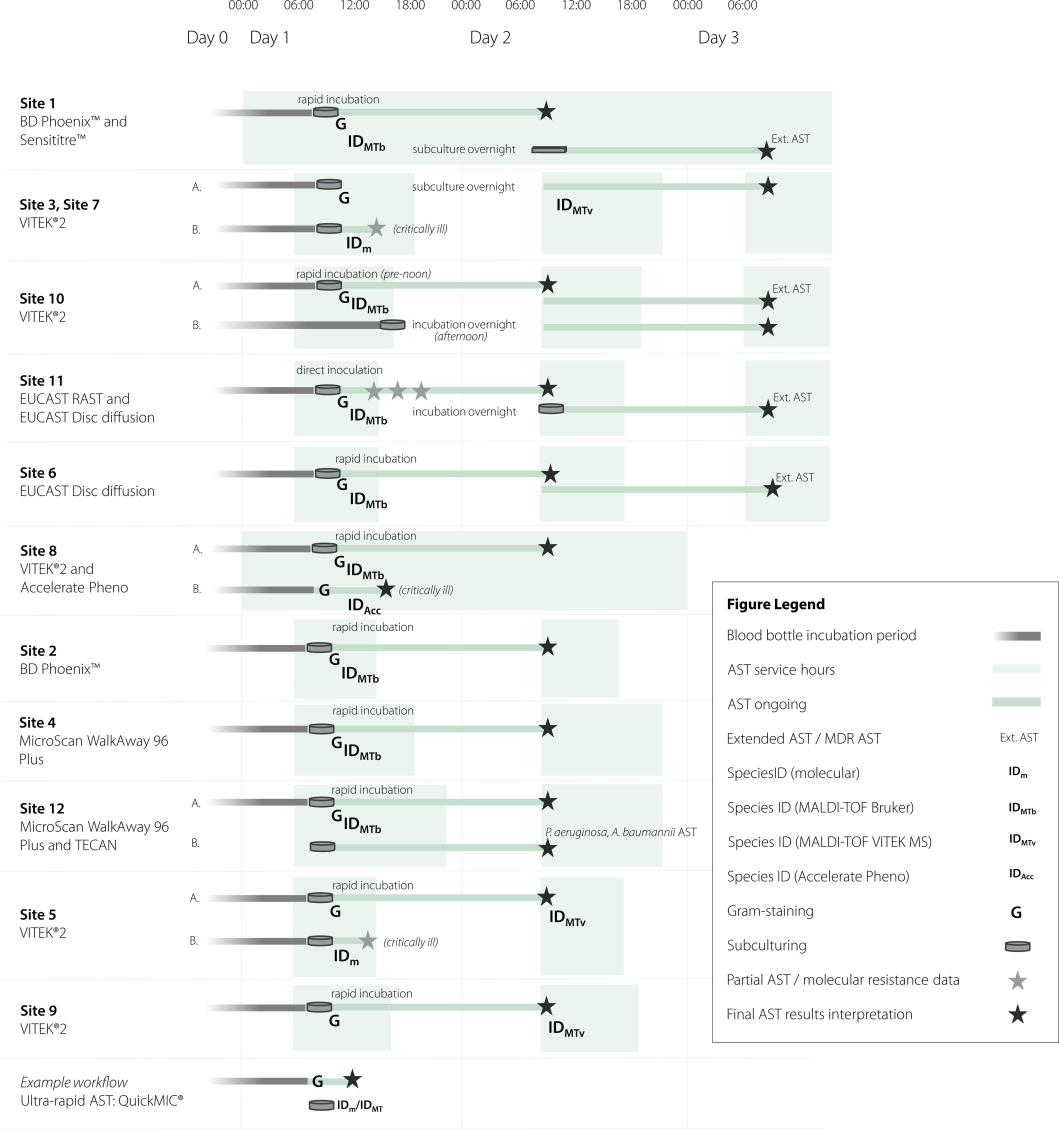
Overview of the workflows at the participating laboratories, from structured workflow analysis. Green fields correspond to AST service times of the laboratories, and the test items are itemized into ID and AST activities. Blood culture in grey, AST in dark green. ID methods are differentiated by type. Some laboratories use parallel workflows, e.g. rapid vs. standard, differentiated by species – these differences are signified by A/B. For a detailed summary of the workflow for each laboratory, see **Supplementary Table S3**.

Workflow analysis results are summarized in **Figure 3** and **Supplementary Text S3**. Multiple laboratories achieved actionable AST results within 2–3 days of initial processing. For these sites, day 1 procedures typically included Gram staining, MALDI-TOF identification, and sub-culturing. This is followed by day 2 where AST is started from the subculture plates, and then day 3 when the AST results are ready. Several laboratories reported shortening this process one day to 1–2 days in total by inoculating the AST method with bacteria from a PBC rapid incubation (~4 h) plate, or by directly inoculating PBC broth into the AST method (off-label protocol). Two laboratories already used a rapid AST method, either EUCAST rapid disc diffusion with multiple read-outs at 4, 6, 8 and 24 h (Site 11) or Accelerate Pheno™ (Site 8) with combined ID + AST results, thus potentially already allowing for same-shift AST. The rapid disc laboratory uses a parallel workflow with a rapid arm setting a limited panel for initial rapid AST and direct from-blood AST to be read next day, followed by an extended panel from the next-day subcultures from the slow arm in case of indications of resistance (**Figure 5**). The laboratory with the Accelerate Pheno™ system similarly reported using parallel slow and rapid workflows, using the standard testing for confirmation. In comparison to the self-reported TATs and previously published TATs for the workflows, QuickMIC results directly from positive blood culture could potentially save ~17 h compared to legacy methods from short-incubation or PBC inoculation; and ~45 h compared to workflows incorporating an overnight subculture before AST start. **Table 4** displays the turnaround-time data used for each workflow comparison.

**Table 4:** TAT (PBC processing to AST result) comparison and estimated savings by using QuickMIC.

Workflow	Self-reported TAT	Published TAT (reference)	Est. saving with QuickMIC
BD Phoenix/subculture	–	48 h (Choi et al., 2017), Positivity to result with 9h avg. PBC to AST start	36 h
BD Phoenix/rapid incubation	24 h	26 h (Maddalena et al., 2023)	21–23 h
Disc diffusion/subculture	48 h	36–47 h (Edmondson et al., 2024)	33–44 h
Disc diffusion/rapid incubation	24 h	36–47 h (Edmondson et al., 2024)	20 - 44h
EUCAST rapid disc/PBC	4, 6, 8 h	4-8h (Soo et al., 2020)	1–5 h
MicroScan WalkAway/subculture	–	48 h (Barman et al., 2010)	45 h
MicroScan WalkAway/rapid incubation	20 h	22.24 h (Sy et al., 2023)	17–19 h
SensiTitre/subculture	48 h	35–48 h (Chapin and Musgnug, 2003)	32–45 h
VITEK2/subculture	48 h	42.5 h (Cavalieri et al., 2019)	39–45 h
VITEK2/rapid incubation	20 h	20.28 h (Soo et al., 2020)	17 h
Accelerate Pheno/PBC	9 h	9.7 h (Truong et al., 2022)	6–7 h

Self-reported TAT is the laboratory-reported average TAT during the workflow analysis. Published TAT is the average TAT for a specific workflow as reported in published literature. Estimated savings by QuickMIC is calculated by subtracting average QuickMIC TTR time from the self-reported and published comparator workflow times from PBC to result.

## 4 Discussion

The results of this multicentre evaluation demonstrate that the QuickMIC rapid AST system provides highly reliable susceptibility results directly from positive blood cultures with a mean time-to-result of just over three hours. This represents a substantial reduction compared to conventional workflows, where actionable AST results are typically available only after one to three days after blood culture positivity. Even in centres employing optimized or rapid protocols, QuickMIC consistently provided results within the same clinical shift, thus potentially enabling earlier therapeutic decision-making. The clinical importance of reducing time-to-result for AST is likely significant. Several studies have shown that delays in administering appropriate antimicrobial therapy in septic patients are associated with increased mortality, longer length of stay, and higher healthcare costs (Kumar et al., 2006; Banerjee and Humphries, 2021). Rapid identification methods, such as MALDI-TOF, have already transformed microbiology workflows by shortening species-level identification times. However, susceptibility testing has remained a bottleneck, with most laboratories constrained by culture-based protocols. QuickMIC addresses this gap by delivering MIC values and categorical interpretations within hours, potentially enabling the treating physician who orders blood cultures to adjust therapy within their shift.

The potential downstream benefits extend beyond individual patients. Early tailoring of therapy allows for both escalation in cases of resistance and de-escalation where broad-spectrum coverage proves unnecessary. Previous studies of rapid AST coupled with stewardship interventions have reported improved appropriateness of therapy, reduced broad-spectrum antibiotic use, and decreased length of stay (Beganovic et al., 2019; Banerjee et al., 2021). Our workflow analysis underscores that while some centres have introduced rapid disc diffusion or existing commercial rapid AST systems, coverage remains limited and workflows heterogeneous. QuickMIC demonstrated consistent performance across sites in both Northern and Southern Europe, including laboratories with high proportions of multidrug-resistant organisms, suggesting broad applicability.

Our study also provides granularity on the performance of the system across species and antibiotics, in comparison to routinely used methods. Time-to-result varied somewhat depending on species and antibacterial agent, with *A. baumannii* representing the slowest-to-report group, though still within clinically useful timeframes. Importantly, categorical agreement rates exceeded 93%, comparable to or better than other rapid AST platforms described in the literature (Charnot-Katsikas et al., 2018; Esse et al., 2023; Ligero-López et al., 2024). The slightly lower essential agreement observed when compared with the Accelerate Pheno system likely reflects the small sample size for this comparator rather than a systematic bias. One important limitation of the study are the uncontrolled conditions which vary significantly between each participating laboratory. This study also did not compare the AST results to reference standard broth microdilution, which means that individual discrepancies may reflect errors in either QuickMIC or the comparator method used at each laboratory, the direction of discrepancy cannot be attributed to either method due to the study design. The workflow analysis however clearly shows that ultra-rapid AST methods such as QuickMIC can significantly decrease TAT of actionable AST results by days and enable same-shift AST results for a majority of samples, even in laboratories with severely constrained AST service hours. The only participating laboratory that did not achieve same-shift AST results in this study only had AST service hours between 8 am to noon (in total 4 h daily), which meant no QuickMIC tests completed within the service window. It is possible that an extension of these service hours by 1–2 hours would be manageable within existing operational budgets, thus allowing same-shift AST using QuickMIC also in this setting.

While these findings are promising, prior studies highlight that implementation of rapid AST is most effective when paired with proactive antimicrobial stewardship and clinician-targeted educational initiatives (Beganovic et al., 2019). Furthermore, the effectiveness of rapid AST depends on reducing the total turnaround time from patient sampling to therapeutic intervention, which is influenced by factors such as blood culture bottle idle time, transport times (from ward to incubator and to the laboratory), laboratory delays, and reporting and stewardship processes (e.g., LIS integration and notification practices). Future prospective studies of QuickMIC should therefore assess not only analytical performance but also patient-centred outcomes, including time to effective therapy, mortality, and antimicrobial utilization.

Health economic evaluations will also be important to establish cost-effectiveness across diverse healthcare settings, particularly given the higher per-test costs associated with rapid AST technologies. These increased upfront costs may be offset if improved clinical outcomes translate into downstream savings elsewhere in the healthcare system. Effective implementation may depend on diagnostic stewardship strategies that prioritize use in critically ill or high-risk patient populations. In addition, increased automation of rapid AST platforms may offer opportunities to reduce personnel requirements and associated labour costs.

Another limitation of the QuickMIC system is the absence of a Gram-positive panel. This reflects practical rather than technical considerations. Few rapid AST systems from positive blood cultures include Gram-positive panels, as syndromic molecular diagnostics already provide actionable resistance information (e.g., *mecA/C*, *vanA/B*) sufficient to guide early therapy. As a result, laboratories may perceive limited added value in performing an additional rapid phenotypic test, particularly given the associated costs. Although this may differ in settings with limited access to syndromic testing, the overall incentive to develop Gram-positive–specific rapid AST panels remains limited.

In summary, this multicentre evaluation confirms that the QuickMIC system delivers accurate and reproducible AST results within 2–4 hours across a diverse range of European hospitals. Compared to traditional workflows requiring 20–48 hours, this represents a substantial reduction in diagnostic delay. Rapid and ultra-rapid AST has the potential to improve patient care in sepsis by enabling timely optimization of antimicrobial therapy, supporting both escalation in resistant infections and de-escalation where broad coverage is unnecessary. Future work should focus on linking rapid AST results to clinical outcomes and on defining implementation strategies that maximize their impact within routine care.

## Data Availability

The original contributions presented in the study are included in the article/**Supplementary Material**. Further inquiries can be directed to the corresponding author.
