# Supplementary material for: Rapid AST in practice – a workflow analysis of the QuickMIC® rapid AST system at multiple clinical laboratories in Europe
Source: Front Cell Infect Microbiol. 2026 May 5;16:1823965. doi: 10.3389/fcimb.2026.1823965 (PMC13183651; doi:10.3389/fcimb.2026.1823965)
Supplement: Supplementary file 1 [file Supplementaryfile1.pdf]

Supplementary data for:

Rapid AST in practice – a workflow analysis of the QuickMIC® rapid AST system at multiple clinical laboratories in Europe

Amanda Åman[1], Victor Englöf[1], Kajsa Knagge[1], Lucas Reibenspies[1] , Maria Avolio[2], Patricia Cabral da Silva[3], Patrizia Cambieri[4], Cristina Costa[2], Adriana Coutinho[5], Monica Dotta[6], Pier Andrea Dusi[6], Danielle Fenwick[7], Giorgia Gregori[8], Sandra Grunewald[9,10], Kerry Laws[7], Giuliana Lo Cascio[8], Paulo Lopes[11], Emma Marrs[7], Luis Morais[12], Joni Mota[12], Loredana Pangaro[13], Micaela Pelagi[13], Sara Petersson[3], Damiano Picicco[6], Tamara Ruegamer[9,10], Sarah Shodunke[7], Annika Wistedt[3], Hanna Woksepp[3,14], Andrea Zappavigna[8], Giuliana Germinario[15], Alessia Cantiani[16], Simone Ambretti[15,16], Cecilia Johansson[1], Anna Olsson[1], Christer Malmberg[1,17]

[1]Gradientech AB, Uppsala, Sweden
[2]S.C. Microbiologia e Virologia U, AOU Città della Salute e della Scienza di Torino, Turin, Italy
[3]Department of Clinical Microbiology, Kalmar County Hospital, Kalmar, Sweden
[4]SC Microbiologia e Virologia, Fondazione IRCCS Policlinico San Matteo, Pavia, Italy
[5]Laboratório de Patologia Clínica, Hospital do Espírito Santo de Évora, Évora, Portugal
[6]SSD Microbiologia, Ospedale di Sanremo, Sanremo, Italy
[7]Microbiology research, Freeman Hospital, Newcastle Upon Tyne, UK
[8]Dipartimento Medicina di Laboratorio, AUSL di Piacenza, Ospedale Guglielmo da saliceto, Piacenza, Italy
[9]Institute for Medical Microbiology, Immunology and Hygiene, University Hospital Cologne
[10]Faculty of Medicine, University of Cologne, Cologne, Germany
[11]Department of Microbiology of Clinical Pathology, Unidade Local de Saúde Gaia Espinho, Vila Nova de Gaia, Portugal
[12]Department of Microbiology of Clinical Pathology, ULSM Tejo, Tomar, Portugal
[13]Department of Clinical Microbiology, Vercelli Hospital, Vercelli, Italy
[14]Department of Biomedical and Clinical Sciences, Linköping University, Linköping, Sweden
[15]Department of Medical and Surgical Sciences, Alma Mater Studiorum - University of Bologna, Bologna, Italy
[16]Microbiology Unit, IRCCS Azienda Ospedaliero Universitaria di Bologna, Bologna, Italy
[17]Department of Medical Sciences, Uppsala University, Uppsala, Sweden.

Correspondence:
Christer Malmberg
christer.malmberg@medsci.uu.se

Table S1: Workflow interview questionnaire

The following guide was used during data collection from laboratory representatives:

**Site Nr:**	**Q:**	**A:**
**Background**	How many hospital beds does the laboratory serve?	
	How many blood-culture sets do you process yearly, approximately? (Set: pair of anaerobe/aerobe bottles)	
	How many ICU departments do you serve?	

	Does your institution have a stewardship team, including clinical microbiologists?	
	Which are the opening hours of the laboratory, weekdays and weekends?	
	When are AST services (AST start, readout, reporting) available during the laboratory opening hours?	
	Which blood culture system is in use at your institution? (Brand, manufacturer)	
	Which AST system is in use at your institution? (Brand, manufacturer)	
	Which bacterial ID methods do you have available?	
**Workflow**	Please describe the handling of blood-culture bottles as they arrive at your laboratory.	
	Please describe the complete workup of positive blood cultures at your laboratory, from Gram-stain to ID and AST steps, including usual timepoints during the workday.	
	Are there any secondary workflows for specific sample types?	
	How are suspected MDR isolates handled onwards?	
	Do you use direct PBC inoculation or short incubation “streak plates” for AST and/or ID?	
	Do you use any rapid AST/ID method today?	
	Do you batch-run or run as samples become positive?	
	How long does it usually take from blood-culture positivity to AST result availability in your laboratory (turnaround-time, TAT)?	

Tables S2: QuickMIC performance per laboratory

EA and CA per site (lab)

Lab	No of MICs	No of categories	No of strains	CA	EA	S	I	R	MiD	MD	VMD	Bias	Time
1	320	320	20	304 (95.0)	310 (96.9)	226	13	81	7 (2.2)	1 (0.3)	8 (2.5)	-3.91	03:11
2	70	70	8	64 (91.4)	59 (84.3)	52	4	14	1 (1.4)	0 (0.0)	5 (7.1)	-26.03	03:04
3	107	105	38	93 (88.6)	93 (86.9)	76	12	17	9 (8.6)	1 (1.0)	2 (1.9)	8.24	03:09
4	367	353	26	320 (90.7)	337 (91.8)	215	9	129	16 (4.5)	8 (2.3)	9 (2.5)	-0.15	03:21
5	94	94	9	90 (95.7)	86 (91.5)	70	5	19	2 (2.1)	1 (1.1)	1 (1.1)	1.10	03:10
6	-	343	47	329 (95.9)	-	308	10	25	4 (1.2)	1 (0.3)	9 (2.6)	-	03:06
7	72	72	9	60 (83.3)	57 (79.2)	47	4	21	5 (6.9)	0 (0.0)	7 (9.7)	-18.02	03:13
8	329	329	24	304 (92.4)	297 (90.3)	235	12	82	10 (3.0)	5 (1.5)	10 (3.0)	-4.93	03:10
9	66	66	8	61 (92.4)	62 (93.9)	57	1	8	1 (1.5)	2 (3.0)	2 (3.0)	0.54	03:04
10	190	190	31	182 (95.8)	184 (96.8)	177	1	12	2 (1.1)	4 (2.1)	2 (1.1)	-0.92	03:05
11	-	210	25	205 (97.6)	-	189	3	18	1 (0.5)	3 (1.4)	1 (0.5)	-	03:06
12	629	628	61	581 (92.5)	573 (91.1)	498	45	85	16 (2.5)	6 (1.0)	25 (4.0)	-6.16	02:58
**Overall**	**2,244**	**2,780**	**306**	**2593 (93.3)**	**2058 (91.7)**	**2,150**	**119**	**511**	**74 (2.7)**	**32 (1.2)**	**81 (2.9)**	**-4.37**	**03:07**

EA and CA per supported species

Species	No of MICs	No of categories	No of strains	CA	EA	S	I	R	MiD	MD	VMD	Bias	Time
ABC*	8	8	1	3 (37.5)	3 (37.5)	1	0	7	2 (25.0)	1 (12.5)	2 (25.0)	-30.00	03:50
Citrobacter spp.	25	25	2	24 (96.0)	24 (96.0)	22	0	3	0 (0.0)	1 (4.0)	0 (0.0)	13.04	03:20
ECC**	140	140	13	131 (93.6)	132 (94.3)	120	2	18	6 (4.3)	3 (2.1)	0 (0.0)	0.26	03:09
E. coli	1134	1525	162	1419 (93.0)	1038 (91.5)	1,232	28	265	43 (2.8)	10 (0.7)	53 (3.5)	-6.92	03:06
Klebsiella spp.	652	748	82	702 (93.9)	610 (93.6)	533	23	192	14 (1.9)	9 (1.2)	23 (3.1)	-5.89	03:06
P. aeruginosa	117	141	23	130 (92.2)	93 (79.5)	67	63	11	8 (5.7)	2 (1.4)	1 (0.7)	8.83	03:08
Proteus spp.	128	146	14	140 (95.9)	120 (93.8)	130	2	14	0 (0.0)	4 (2.7)	2 (1.4)	2.10	03:19
Serratia spp.	40	47	9	44 (93.6)	38 (95.0)	45	1	1	1 (2.1)	2 (4.3)	0 (0.0)	7.50	03:18

*Acinetobacter baumannii-complex, ** Enterobacter cloacae-complex

EA and CA per unsupported species

Species	No of MICs	No of categories	No of strains	CA	EA	S	I	R	MiD	MD	VMD	Bias	Time
Morganella spp.	5	5	1	3 (60.0)	2 (40.0)	2	0	3	0 (0.0)	0 (0.0)	2 (40.0)	-41.67	03:18
P. stuartii	10	10	1	8 (80.0)	9 (90.0)	3	1	6	1 (10.0)	1 (10.0)	0 (0.0)	46.03	03:44
Salmonella spp.	0	3	1	3 (100.0)	-	3	0	0	0 (0.0)	0 (0.0)	0 (0.0)	-	02:50

EA and CA per antibiotic on the QuickMIC GN panel

AB	No of MICs	No of categories	No of strains	CA	EA	S	I	R	MiD	MD	VMD	Bias	Time
AMI	242	309	288	296 (95.8)	226 (93.4)	289	0	20	0 (0.0)	2 (0.6)	11 (3.6)	8.07	02:28
CEP	203	203	269	170 (83.7)	176 (86.7)	109	30	64	28 (13.8)	4 (2.0)	1 (0.5)	-2.13	03:15
CIP	216	288	270	259 (89.9)	199 (92.1)	169	28	91	23 (8.0)	4 (1.4)	2 (0.7)	-10.82	03:24
COL	149	149	238	141 (94.6)	132 (88.6)	135	0	14	0 (0.0)	3 (2.0)	5 (3.4)	-20.65	02:53
CTA	154	220	240	215 (97.7)	150 (97.4)	160	1	59	2 (0.9)	3 (1.4)	0 (0.0)	4.25	03:32
CTV	161	184	268	177 (96.2)	141 (87.6)	172	0	12	0 (0.0)	3 (1.6)	4 (2.2)	-3.28	02:56
CTZ	220	289	272	275 (95.2)	209 (95.0)	178	32	79	11 (3.8)	1 (0.3)	2 (0.7)	7.81	03:26
GEN	222	244	259	232 (95.1)	208 (93.7)	205	0	39	0 (0.0)	7 (2.9)	5 (2.0)	-0.64	03:09
MER	237	303	254	291 (96.0)	224 (94.5)	278	7	18	8 (2.6)	0 (0.0)	4 (1.3)	-42.13	03:13
PIT	243	313	269	276 (88.2)	207 (85.2)	222	21	70	2 (0.6)	3 (1.0)	32 (10.2)	-14.20	03:16
TIG	80	95	152	88 (92.6)	79 (98.8)	87	0	8	0 (0.0)	0 (0.0)	7 (7.4)	-2.20	03:12
TOB	117	183	240	173 (94.5)	107 (91.5)	146	0	37	0 (0.0)	2 (1.1)	8 (4.4)	-7.66	02:52
**Overall**	**2,244**	**2,780**	**306**	**2593 (93.3)**	**2058 (91.7)**	**2,150**	**119**	**511**	**74 (2.7)**	**32 (1.2)**	**81 (2.9)**	**-4.37**	**03:07**

Table S3: Rates of MDR bacteria per laboratory

WT: Wildtype (no resistance), RES: Any resistance, MDR: Resistant to ≥3 antibiotic groups

Site	WT	RES	MDR
1	30.0	70.0	35.0
2	33.3	66.7	33.3
3	63.9	36.1	0.0
4	26.9	73.1	34.6
5	44.4	55.6	22.2
6	72.9	27.1	6.2
7	44.4	55.6	55.6
8	25.0	75.0	45.8
9	62.5	37.5	12.5
10	80.6	19.4	3.2
11	80.0	20.0	8.0
12	54.1	45.9	13.1
Overall	55.9	44.1	17.0

Text S4. Structured workflow analysis – summary of the laboratory survey response

Site 1: The laboratory is open 24/7 and blood culture bottles are incubated throughout the day. When a blood culture signals positive Gram-staining is performed and rapid incubation (4h) initiates AST using BD Phoenix. The results were reported the following day. If a multi drug-resistant phenotype is identified, testing using Sensititre™ is initiated on day two.

Site 2: Blood bottles are incubated 24/7. The laboratory is open 08-15 Monday-Saturday and AST is performed in batch once a day (8-13) on positive blood cultures available in the morning, using the BD Phoenix system**.** AST and ID is facilitated by rapid subculturing.

Site 3. Blood bottles are incubated 24/7. The laboratory is open 07.30-20 Monday-Friday, 07.30-14.30 on Saturday and Sunday. A. Positive blood cultures are subcultured as they turned positive during opening hours. AST is initiated on day two and results are interpreted the following day. B. For critically ill patients or with indications of sepsis molecular testing using BIOFIRE® FILMARRAY® is initiated day one and reported as a preliminary result.

Site 4: Blood bottles are incubated during opening hours of the laboratory Monday-Friday 8-20. Blood cultures are handled as they turn positive during the day. AST using MicroScan WalkAway Plus can be initiated after rapid subculturing and results are reported the following day.

Site 5: The laboratory is open Monday-Friday 8-16 and Saturday -Sunday 8-12. Blood cultures are incubated as soon as they reach the lab during opening hours. A. Blood cultures that signal positive before noon are subjected to Gram-stain and rapid incubation to initiate AST. B. For critically ill patients molecular testing using BIOFIRE® FILMARRAY® is initiated day one.

Site 6: Blood bottles are incubated 24/7. The laboratory is open 08-17 Monday-Friday and 08-13.30 on weekends. Blood cultures that signals positive before noon are subcultured rapidly and tested with EUCAST disc diffusion when there is enough material on the subculture, otherwise AST is started next day. The results are interpreted the following day according to EUCAST guidelines.

Site 7: Blood bottles are incubated 24/7. The routine microbiology laboratory is open 08-20 from Monday to Friday and 08-15 on Saturdays and the Emergency Lab 24/7. A. AST is performed from Monday to Saturdays using VITEK2 and positive blood cultures are subcultured in batch to initiate analysis the following morning. B. Molecular testing using BIOFIRE® FILMARRAY® is also initiated if the blood culture came from the paediatric ward or there are clinical indications for sepsis/septic shock or severe infection.

Site 8: The laboratory is open 24/7 and blood cultures are incubated whenever available. When a blood culture signals positive Gram-staining is performed. A: AST and species identification is facilitated by rapid subculturing. VITEK2 is used for AST and Maldi-Tof (Bruker) for ID. The AST results are interpreted on the following day. B: Accelerate Pheno rapid ID+AST is used for critically ill patients.

Site 9: Blood bottles are incubated when received by the laboratory. The laboratory is open 08-17 daily and AST using VITEK2 is performed during opening hours after rapid incubation for subculture.

Site 10: Blood bottles are incubated during opening hours. A. For blood cultures that signals positive before noon, rapid incubation is performed and after 4 hours, VITEK2 testing is initiated. The AST results are interpreted the following day. B. Blood cultures, which turn positive in the afternoon or evening, are subject to overnight incubation to initiate VITEK2 testing on day two. These results are interpreted the following day. For multi-drug-resistant phenotypes, extended AST testing is done.

Site 11: Blood bottles are incubated as received during core hours 08.30-20.00 Monday-Friday, 07.30-12.30 on Saturday and Sunday. Those received overnight are incubated the following morning. The emergency lab is open 24/7. AST is performed on all positive blood cultures during core hours as they flag positive, using EUCAST Disc diffusion. EUCAST RAST is carried out on all samples containing suitable Gram-negative isolates that are identified before 10.00 each day.

Site 12: The laboratory is open 24/7 for bottles incubation and follow-up analysis of positive blood cultures, including gram-staining, subculture, rapid species identification by MALDI-TOF and AST preparation (both performed on growth obtained after short incubation). All the results are reported when available (from 8 to 20 for AST). AST is performed with MicroScan WalkAway (A) or the ASTroID AST automation system specifically for *Pseudomonas aeruginosa* (B).

Figure S5. Simulated analysis of “same shift” results by longer average TTR

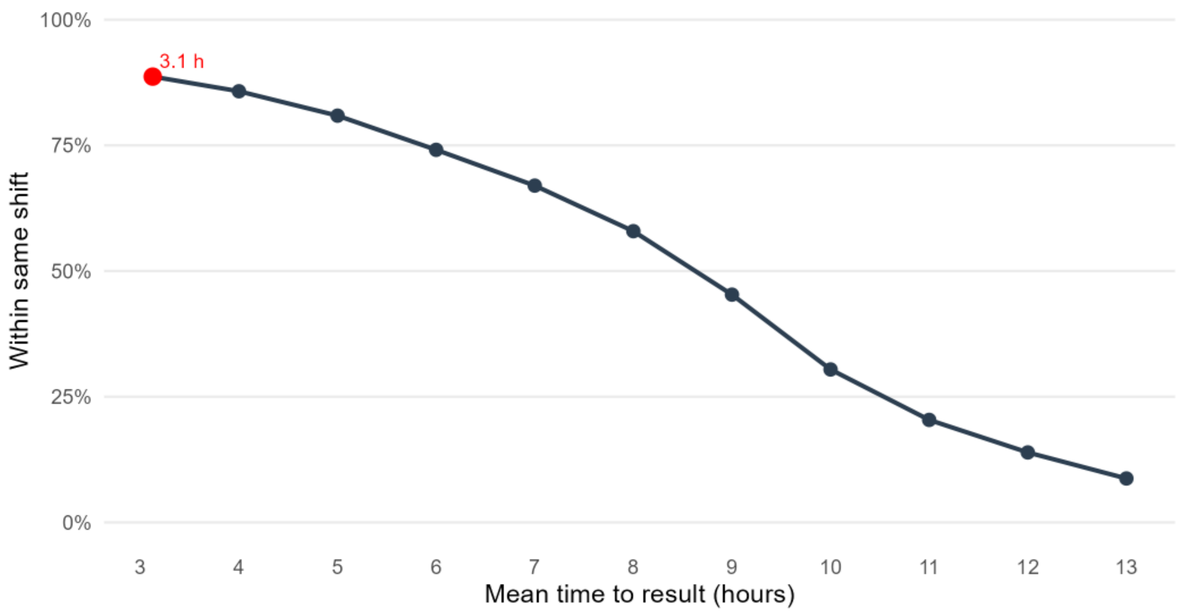

The analysis shows the percentage of samples which would have been reportable during the AST service hours of the laboratories in the study, should the average TTR have been longer. Times up until 13h average TTR were simulated. The red dot indicates the QuickMIC average TTR in the present study.
